# 1830. Social determinants of cardiovascular disease risk factor control among people living with HIV

**DOI:** 10.1093/ofid/ofad500.1659

**Published:** 2023-11-27

**Authors:** Melissa Klein Cutshaw, Kelley Jones, Chris Longenecker, Nwora Lance Okeke

**Affiliations:** Duke University, Durham, North Carolina; Duke University, Durham, North Carolina; University of Washington, Seattle, Washington; Duke University, Durham, North Carolina

## Abstract

**Background:**

HIV is associated with an elevated risk of cardiovascular disease, yet hypertension and high cholesterol are often sub-optimally treated in people with HIV. The role of racial disparities and social determinants of health in the management of these cardiovascular risk factors is poorly understood.

Average systolic blood pressures vs TIS score (hypertension treatment intensity), by Black race

**Figure:**
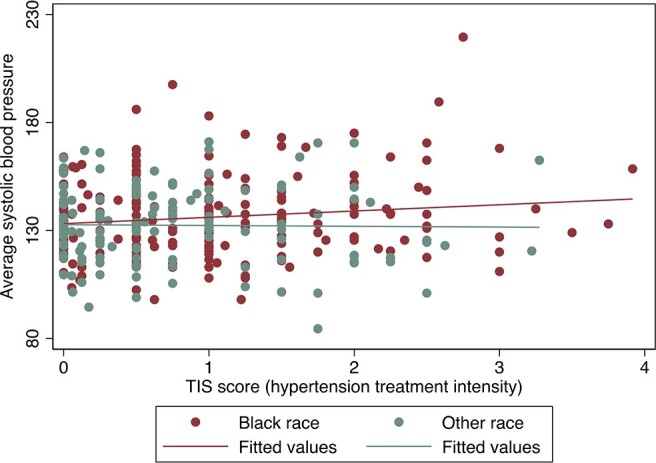

TIS (therapeutic intensity score) was calculated by dividing the patient’s antihypertensive medication dosage by the maximum daily dosage approved by the US Food and Drug Administration, then summing this proportion for each antihypertensive medication prescribed. For example, if a patient was prescribed two antihypertensive medications each at 75% of the maximum approved dosage, the TIS score would be calculated as 1.5 (0.75 + 0.75).

**Methods:**

We performed a cross-sectional analysis of adult patients with HIV among three academic medical centers in the United States. We considered various social determinants of health including race, sex, measures of economic stability, education level, health insurance, health literacy, social deprivation index by zip code, and measures of psychosocial stress. We examined a hybridized outcome of risk factor control (poor or good) and treatment intensity (minimal or maximal) for two key cardiovascular risk factors, hypertension and hyperlipidemia. Multinomial logistic regressions were performed to identify factors associated with cardiovascular risk factor control and intensity, with “good control, maximal treatment” used as the reference group.

**Results:**

Overall, 371 persons were included in the analysis. Participants were 23.5% female, 59.6% Black, 7.3% Hispanic/Latino, with a median age of 59 (interquartile range 53-64). Among study participants with hypertension, Black participants were more likely to have poor control despite maximal treatment (relative risk ratio [RRR] 2.95, 95% CI: 1.30-6.66). In our hyperlipidemia analysis, having the highest quartile of perceived stress was more common among participants with poor than good cholesterol control (27.0% vs 15.9%, p=0.032). Compared to participants with good control and maximal treatment of high cholesterol, participants with the highest quartile of perceived stress were more likely to have poor control with minimal (RRR: 2.80, 95% CI: 1.02-7.63) or maximal (RRR: 3.64, 95% CI: 1.20-11.1) treatment.

**Conclusion:**

Race and social determinants of health, including perceived stress, appear to influence cardiovascular disease risk factors among persons with HIV.

**Disclosures:**

**Nwora Lance Okeke, MD MPH**, Gilead Sciences: Advisor/Consultant

